# Antimicrobial Resistance and Its Drivers—A Review

**DOI:** 10.3390/antibiotics11101362

**Published:** 2022-10-05

**Authors:** Mohammad Irfan, Alhomidi Almotiri, Zeyad Abdullah AlZeyadi

**Affiliations:** Department of Clinical Laboratory Sciences, College of Applied Medical Sciences, Shaqra University, Ad Dawadmi 17464, Saudi Arabia

**Keywords:** antibiotics, antimicrobial resistance, environmental drivers, biocides, public health, spread of resistance

## Abstract

Antimicrobial resistance (AMR) is a critical issue in health care in terms of mortality, quality of services, and financial damage. In the battle against AMR, it is crucial to recognize the impacts of all four domains, namely, mankind, livestock, agriculture, and the ecosystem. Many sociocultural and financial practices that are widespread in the world have made resistance management extremely complicated. Several pathways, including hospital effluent, agricultural waste, and wastewater treatment facilities, have been identified as potential routes for the spread of resistant bacteria and their resistance genes in soil and surrounding ecosystems. The overuse of uncontrolled antibiotics and improper treatment and recycled wastewater are among the contributors to AMR. Health-care organizations have begun to address AMR, although they are currently in the early stages. In this review, we provide a brief overview of AMR development processes, the worldwide burden and drivers of AMR, current knowledge gaps, monitoring methodologies, and global mitigation measures in the development and spread of AMR in the environment.

## 1. Introduction

We continue to face an antimicrobial-resistance challenge from drug-resistant microorganisms that are continuously developing novel resistance mechanisms. This puts our ability to cure common ailments in jeopardy. Numerous bacteria are acquiring resistance to a wide variety of drugs, and these germs cause infections that are not treatable with the currently available antimicrobials. This is perhaps the most troubling part of the issue [1]. Although genes carrying AMR mechanisms were discovered in bacteria long before antibiotics have been introduced to the clinic [2], it is difficult to precisely specify what is the main selective pressure that causes the emergence of resistance, but the misuse of antimicrobials in the medical, veterinary, and agricultural sectors is usually considered the key reason [3].

Resistance is driven by a variety of factors that are complex, diverse, and cross-sectoral in nature. In addition to sharing the same habitat, humans and animals also share several infectious diseases that may have started in animals at some point during their evolution into humans [4,5,6]. There are several ways in which resistant bacteria can spread across international borders, either through direct exposure or through the food chain and environmental transmission [3,4]. The occurrence of AMR organisms and antimicrobial resistance genes (ARGs) are regularly reported from a variety of sources, including people, animals, food, plants, and the surrounding environment [1].

Significant changes in food intake as a consequence of increased population density may have been one of the driving forces behind the resistance. This is in addition to the global sanitation and water pollution concerns posed by sewage, manure runoff, and waste generated by pharmaceutical manufacturing and hospitals. AMR is expected to inflict an estimated 10 million losses by 2050 [7], and some other analyses suggested that AMR would cost the worldwide market USD100 trillion within the equivalent time frame [8].

Identifying and regulating the importance of environmental factors in the genesis and progression of AMR is crucial to effectively combat AMR globally. Identifying and addressing the variables that contribute to AMR in the population will be crucial to develop and implement effective prevention and management strategies for AMR, as well as to monitor the environmental sources of AMR. This study highlights the causes of antibiotic resistance, as well as existing knowledge gaps, surveillance approaches, and international mitigation efforts.

## 2. Antimicrobial Resistance: A Worldwide Public Health Emergency

In a recent estimate, about 3.57 million of 4.95 million deaths worldwide were associated with antimicrobial resistance in 2019 [9], higher than many other well-known causes of mortality, including malaria and HIV/AIDS. According to the study, the worldwide burden of AMR is significantly higher than the 700,000 deaths per year projected by the World Health Organization (WHO) and the United Nations [10]. There had been a previous projection that antimicrobial resistance would kill 10 million people per year by 2050 [7], but the truth has now been revealed that we are closer to that figure than we thought. Globally, AMR trends vary significantly among various countries and often face different principal challenges. However, in contradiction to various health-care concerns, AMR is a challenge that concerns each nation, regardless of the financial level.

In the past, the continuous discovery of new antibiotic classes helped reduce the burden of infectious diseases; however, pathogenic bacteria were able to develop resistance to these new compounds within a few years [11]. For example, within a year of approval of the aminoglycoside streptomycin by the US FDA for the treatment of TB, resistance was reported in some patients [11]. Carbapenems and colistin are antibiotic classes that are used as a last resort to treat infections caused by bacteria that produce a wide spectrum of β-lactamases or are multidrug-resistant [12]. In all corners of the world, there has been an increase in Enterobacteriaceae resistance—one of the WHO priority pathogens—to carbapenems and colistin [13,14,15]. A study by Hasunna et al. reported alarmingly high resistance rates of almost 83.35% among extensively drug-resistant (XDR) *Klebsiella pneumoniae* in neonatal sepsis [16]. Furthermore, almost 500,000 new cases of rifampicin-resistant tuberculosis (RR-TB) were identified around the world in 2018, with a majority of these cases showing multidrug resistance (MDR) [1], and the persistence of antibiotic tolerance can worsen medical conditions related to diseases such as HIV, malignancies, and malaria.

The risks of AMR are projected to increase dramatically as the routine antibiotic treatment regimen becomes obsolete, possibly leading to scenarios in which terminally ill patients need palliative care, but the medications provided are no longer clinically effective [17] and hospital ICUs become hot zones for nosocomial MDR pathogens. Finally, AMR-related epidemics could lead to a 2% to 3.5% decline in global total GDP by 2050, as anticipated in various studies to cost USD60–100 trillion [8,18,19].

## 3. Antimicrobial Resistance: Potential Threats

From the early utilization of prontosil and the discovery of penicillin by Alexander Fleming in 1928 through the close of the previous century, remarkable breakthroughs have been achieved in the study and manufacture of anti-infective drugs, contributing to the major advancement of modern medicine [20]. However, the current decline in antibiotic R&D from major pharmaceutical companies, compounded by the misuse of antibiotics and the excessive consumption and exploitation of antimicrobials as animal growth promoters and in agricultural settings for animal feed, has resulted in pressure that has led to continued exposure and increased tolerance to antimicrobials. AMR poses a significant risk to public health [21]. The bacteria associated with animals can potentially behave as a repository of antibiotic-resistant determinants that could be carried over to humans [22]. In the growth and transmission of AMR, there is a complicated interaction between people, livestock, and the ecosystem [23,24]. According to the latest analysis of scientific research on antibiotic use in agricultural production, only seven analyses (5% of the total screened publications) contended that there had been little correlation between antibiotic intake in animals and human resistance, while 100 (72%) managed to find a significance [25]. The degree to which this transition from animal to person also occurs is of immense importance, with considerable concern for the community and the well-being of animals [23,26,27].

The first incidents of tolerance to antibiotics in food-producing animals were observed in 1951, following the administration of streptomycin to turkeys [28]. Recently and more significantly, the transmissible *mcr-1* gene conferring resistance to the nephrotoxic last-line antibiotic drug colistin was found in *Escherichia coli* isolates from unprocessed meat, livestock, and infected people [29]. Within a span of only three months after its discovery, the *mcr-1* gene was identified in more than twenty different nations [30,31]. Furthermore, pan-drug Gram-negative bacilli (GNB) isolates are being increasingly reported around the world [32]. *Staphylococcus aureus* associated with livestock methicillin (LA-MRSA) was first described in 2005 [33]. As a consequence of the acquisition of enterotoxin genes and other virulence factors, such as the Panton–Valentine leukocidin (pvl) gene, LA-MRSA poses a risk to people [34,35]. Concerns have been raised about the transmission of LA-MRSA from colonized animals, such as pigs, cattle, and poultry, to humans through direct contact with animals, environmental pollution, or meat handling and consumption [36]. More importantly, the rise of vancomycin-resistance enterococci (VRE) presents a serious threat [37,38]. Through both inherent and acquired mechanisms, enterococci develop resistance to various antibiotics [38,39]. Enterococci share genetic material between themselves or with other genera, and it has been shown that vancomycin-resistant *Staphylococcus* spp. (VRSA) have acquired *vanA* (the gene conferring resistance to vancomycin) from animal-associated VRE [40]. Enterococcal infections resistant to vancomycin may also develop resistance to other clinically important antibiotics, such as daptomycin and linezolid, contributing to the rise of AMR against clinically relevant drugs [41,42].

A *Salmonella typhimurium* strain harboring *bla*_NDM-5_ has been discovered in commercial pork for the first time in Jiangsu province in China [43]. Diaz et al. reported an epidemic of *Salmonella enteritidis* resistant to nalidixic acid and showed that the source of contamination was chicken sandwiches [44]. A study carried out in eight Chinese provinces in 2010 found veterinary antibiotic metabolites such as ciprofloxacin, enrofloxacin, oxytetracycline, and chlortetracycline in the feeding of cattle manure [45]. Otto et al. [46] observed that the incidence of ceftiofur-resistant *Salmonella enterica* serovar Heidelberg in Québec and Ontario was significantly related to chicken intake and inappropriate antibiotic use in poultry animals. Furthermore, *Campylobacter jejuni* cultured from commercial chicken items has been shown to be substantially related to clinical human samples from the United States [47]. These examples and more provide compelling evidence that extensive animal farming is one of the major routes for AMR spread.

## 4. Mechanisms and Drivers Contributing to the Spread of AMR

### 4.1. Mechanism of AMR

**Figure 1:**
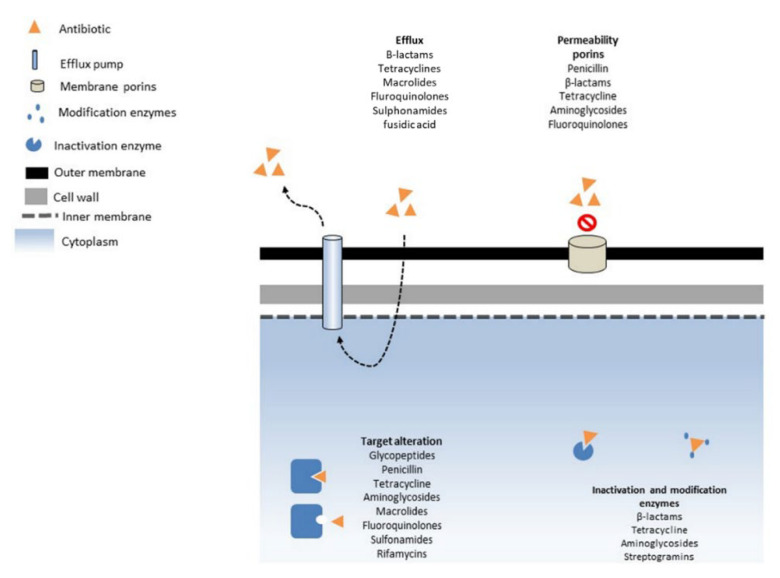
Mechanisms of antibiotic resistance in bacteria. Bacterial cell membrane barriers and membrane protein can prevent antibiotics uptake, or bacteria can reduce intracellular concentration by efflux pumps. Also, antibiotic targets can be modified to reduce affinity. Finally, some antibiotics can be deactivated by specialized enzymes like β-lactamases. Sometimes, several mechanisms of resistance act in concert to provide high-level resistance.

The exposure of bacteria to subinhibitory concentrations of antibiotic drugs is one of the primary causes of antimicrobial resistance (AMR), which is mainly caused by the improper use of antibiotics in clinical and agricultural settings [48,49]. Antibiotic-resistant bacteria use a variety of mechanisms, such as antibiotic inactivation by enzyme breakdown or a change in the enzymatic scaffold; the expression of efflux pumps maintains intracellular antibiotic concentrations below inhibitory levels; alterations to the antibiotic’s intended target; and modifications of the cell membrane’s permeability (Figure 1) [50]. Enzymatic degradation or modification of the antibiotic scaffold is one resistance mechanism that renders the drug ineffective. The classic examples of these enzymes are the β-lactamases and TetX antibiotic-modifying enzymes [51,52,53,54]. Antibiotic resistance can also be developed by protecting, modifying, or overexpressing the intended target. Altering the cell-wall PBP to overcome β-lactam antibiotic activity is the best-known example: VRE use this strategy by enzymatically modifying the peptidoglycan, which reduces the target’s affinity for vancomycin [41]. In addition, two other resistance mechanisms include the use of efflux pumps or changes in membrane permeability to prevent the antibiotics from entering the bacterial cells. Bacteria produce either a multidrug efflux pump [55] or an antibiotic-specific exporter, such as tetracycline efflux pumps [56], to keep antibiotic concentrations within the cell at subinhibitory levels. On the contrary, few bacteria decrease porin expression or produce a more selective porin variant to prevent antibiotics from entering the cell by lowering membrane or wall permeability [57]. Some bacteria can develop extensive resistance by using many complementary mechanisms. Clinical isolates of *Enterobacter cloacae* develop high-level resistance to carbapenems as a result of a porin mutation that reduces carbapenem absorption and increases the synthesis of a chromosomal β-lactamases [58].

The development of antibiotic resistance in bacteria often occurs through vertical and horizontal gene transfer. A mechanism known as “vertical gene transfer” passes genetic information, including any mutations, from one generation to the next within a family. Horizontal gene transfer (HGT) is the primary mechanism through which antibiotic-resistance genes are rapidly disseminated across several bacterial species [59]. Resistant bacteria are able to flourish and spread across the environment because of a number of distinctive characteristics that they possess. Native bacteria in the environment serve as a reservoir for ARGs, which can then be passed on to pathogens through HGT [60,61]. HGT can occur through transformation, transduction, and conjugation, as well as other mechanisms [62]. The mechanisms of HGT are well known under ideal conditions, but in environments with chemical stressors, such as antibiotics and biocides, they are less understood. Subinhibitory and subtherapeutic antibiotic doses would promote the development of resistance by microbes to the drug by triggering natural selection in microbes with marginally higher tolerance to antibiotics through mutation or horizontal gene transfer. Compounded by the quick development of antibiotic resistance at the molecular level, the AMR situation is deteriorating due to a lack of financial motivation, difficulties in clinical research, and scientific innovation gaps. The development of novel antibiotics has hit a roadblock [63], and new antibiotics introduced into clinical use are scarce.

### 4.2. The Excessive Use of Antibiotics

**Figure 2:**
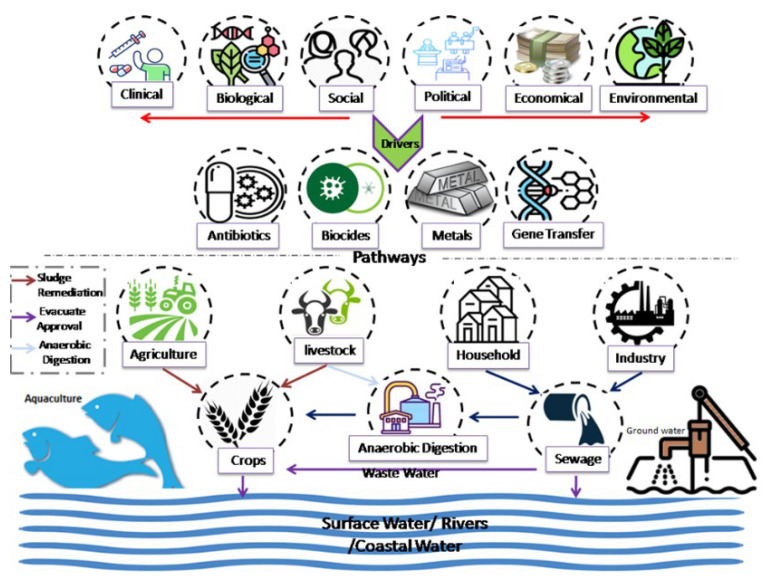
AMR is a global problem that impacts not only people but also animals and ecosystems, both domestic and nondomestic. It is fueled by clinical, biological, social, political, economic, and environmental factors. The presence of AMR bacteria in the environment is a consequence of all of these drivers.

The overuse and misuse of existing antimicrobials have accelerated the emergence of AMR at an alarming rate (Figure 2). Between 2000 and 2015, global antibiotic use increased by 65% [64]. The consumption of antimicrobials per thousand people in China increased from 1910 (defined daily doses) DDDs to 3660 DDDs between 2000 and 2015, while in Brazil this number increased from 2535 DDDs to 6763 DDDs, and in Saudi Arabia it doubled from 5647 DDDs to 10934 DDDs [65]. The use and misuse of antimicrobials are more common in some settings, such as general and acute care wards, and among professionals treating newborns and children, as well as certain diseases or syndromes. According to previous research, incorrect antibiotic use in primary care is up to 55% in South Africa [66], 88% in Pakistan [67], 61% in China [68], and 15.4% in Canada [69], and up to 60% of antibiotic prescriptions given to people with acute respiratory tract infections in primary care settings in Louisiana, USA were clinically inappropriate [70]. This made the initial therapy ineffective, allowing the selection of AMR bacteria to spread and proliferate. Acute bronchitis, sinusitis, and middle-ear infections are other diseases that are often incorrectly treated with the wrong type or dose of antibiotics [69]. Finally, there was an increase in the prescription of antibiotic prophylaxis as part of the treatment regimen for infected patients during the COVID-19 pandemic, and many studies have reported an increase in AMR pathogens [71]. Overuse and misuse of antibiotics will increase the probability that AMR develops over time, specifically bacteria in the WHO priority pathogen group [72].

Since the 1950s, there has been a steady increase in the demand for meat and dairy products around the world, leading to an increase in the use of antimicrobial drugs in agriculture [73], since they are used not only as medications but also as growth stimulants, prophylactics, and metaphylaxis. This excessive use contributed to the development of antibiotic-resistant bacteria and their associated AMR genes, which have the potential to spread and be passed on to people through the food chain. Around 85,330 tons of veterinary antibiotics were consumed around the world in 2017 [74]. The use of antibiotics in food-producing animals is expected to increase by 11.5% by 2030 [75]. The use of antibacterial drugs to stimulate development has been prohibited in the European Union (EU) since 2003 [76], and the FDA eventually outlawed the use of antibiotics in cattle without a veterinarian’s prescription in 2012 [77]. In 2019, there were still 26 nations out of a total of 160 that used antibiotics in agricultural settings as growth promoters [74]. According to Ejo et al. [78], 5.5% of raw meat, eggs, milk, minced meat, and burger samples in Ethiopia had salmonellosis. The isolates were 47.6% resistant to ampicillin, tetracycline, and sulfamethoxazole–trimethoprim. Furthermore, Rasheed et al. [79] found 14.7% multidrug-resistant *E. coli* on vegetable salads, fresh milk, raw chicken and beef, and on the surface of raw eggs. Four percent of these samples had extended-spectrum β-lactamase activity.

### 4.3. Biocides

Biocides are commonly used antimicrobial agents in health-care facilities, beauty brands, residential disinfecting products, wipes and home furnishing additives, farmyards for applications, including wheel and foot rinses, and a wide range of factory methods, such as with the grasp of fouling and souring of piping systems along with oil wells (e.g., hydraulic fracturing) [80]. There are several widely accepted biocides, including ethanol, formaldehyde, chlorhexidine, triclosan, and quaterium ammonium compounds (QACs), alkyldimethyl-benzyl ammonium chloride (ADBAC), stearalkonium chloride, isothiazolium-benzalkonium chloride, cetrimonium chloride/bromide and cetylpyridinium chloride] [81].

In contrast to antibiotics, biocides generally have several target sites [82]. The efficiency of a biocide is proportional to its concentration: at low levels, it may have only a limited impact [83]. If an antibiotic operates only at a single target location and the cell’s adaptive mechanism stops it from reaching that location, resistance may develop. The mechanism of action of biocides, particularly at low or subinhibitory doses, is still poorly understood.

The general mechanism of action of the biocide may be described by the structure of the bacteria against which it is most effective. There are three layers of interaction: cellular components outside the cell, with the cytoplasmic membrane, and with cytoplasmic components. To develop its antimicrobial effect, a biocide can interact with bacterial cells at one or all three levels [84]. The bacterial response to biocides is primarily determined by the properties of the chemical agent and the kind of organism treated. Antibacterial activity may also be significantly affected by parameters such as contact temperature, ambient pH, and the presence of organic matter [85].

The resistance processes to biocides depend on the structural properties of the bacteria, which may be inherent or acquired. Intrinsic resistance is an inherent chromosome-regulated characteristic of a bacterial cell that allows it to evade a biocide. Bacteria that produce endospores are significantly more resistant to biocides such as clostridium and bacillus. Furthermore, physiological adaptation is believed to contribute significantly to increased bacterial resistance to biocides, such as in the case of *Pseudomonas* resistance to certain concentrations of alcohols [86].

Similar to antibiotic resistance, the development of biocide resistance is mutational or acquired [87,88,89]. *E. coli*, *Proteus mirabilis*, *Pseudomonas aeruginosa*, and *S. marcescens* are examples of bacteria resistant to chlorhexidine, a common hospital disinfectant [90,91]. Plasmids in Enterobacteriaceae can include genes for antibiotic resistance and, in certain cases, protection against mercury, organomercury, and other cations and some anions [92]. Many bacterial species have been shown to possess biocidal resistance genes (BRGs), such as the qacE and qacA/B genes found in the Enterobacteriaceae family and *Pseudomonas* and the qacA/B genes found in *S. aureus*, which confer resistance to QACs [93]. Resistance to silver salts mediated by plasmids is especially relevant in hospitals, where silver nitrate and silver sulfadiazine can be used topically to treat severe burns and prevent infection.

A small number of biocides have been shown to be inactivated, and this applies only to a subset of the total number of biocides. As a result of the abundance of cellular targets that biocides can attack, protection is most likely and often derived from variations in the permeability of the cell membrane or enhanced biocide outflow [84]. The Biocidal Products Regulation (EU) 528/2012 supervises commercialization, consumption, and management and specifically governs its use in veterinarian medicine in Europe [94]. Throughout 1992–2007, the world’s economy for biocides expanded by 40% [95]. Wastewater treatment facilities are an important entry point for biocides into the environment. Resistant microbes are more likely to develop when biocides are diluted and discharged into the environment.

### 4.4. Metals

Residential effluent, sanitary sewers, industrial effluent and emissions, and traffic-related output are the main metropolitan sources of heavy-metal ions [96]. It has been reported that various food products, commodities for residential and commercial use, supplies for hospitals, fabrics, and toiletry products for cleaning purposes are extensively employed with the use of metal nanoparticles. Metals such as lead, copper, zinc, cadmium, and arsenic have been used as livestock health stimulants and vitamin supplements. Various metals are also contributing to traces of metals in agronomic soil. If one arranges the factors that contribute to metal deposition in farmland in terms of the order of higher to lower concentrations, these include farm animal manure as the main contributor followed by sludge manure that incorporates nitrogen, phosphorus, potash, and lime, exterior surroundings, and agricultural water as the lowest contributors to heavy metals [97].

Antibiotic- and metal-resistance mechanisms have several structural and functional similarities, such as drug and metal sequestration, reduced membrane permeability, drug and metal efflux, drug and metal alteration, and alteration of cellular targets [98]. There is a direct association between the affinity of metal for thiol compounds and its toxicity to microorganisms [99]. Metal speciation has been studied for its potential to predict metal mobility and toxicity in soils, sediments, and aquatic systems [100,101,102]. Despite this, the relationship between metal bioavailability, speciation, and the selection of resistant gene variations has not been studied [103].

Heavy metals in the influent can have harmful consequences, which can affect biological wastewater treatment processes [104,105], such as nitrification, denitrification, and organic removal [106,107,108]. Several factors influence the toxicity of heavy metals in wastewater, including the solubility of metal ions, pH, the concentration of sludge, the pollution load, and the species and concentration of heavy metals [109]. Heavy metals are toxic to cells because they destroy membranes, interfere with normal physiological processes, and denature proteins and DNA [110,111]. There is evidence that heavy metals can disrupt osmotic equilibrium and the oxidative phosphorylation pathway.

It is not clear why certain microorganisms are more effective than others in removing metals from the environment; however, this might be due to differences in genetics or in the reduction medium [112]. Because climate favors the selection of metals and antibiotic resistance, metals can cause antibiotic resistance in the natural environment [113]. Tolerance to antibiotics is elevated as a result of coselection processes triggered by heavy-metal contamination and subsequent coregulation of resistance genes. It is well established that heavy-metal ions alter antibiotic sensitivity and coregulate genes involved in antibiotic resistance [98]. ARGs are generally located on plasmids, and it has been shown that bacteria with metal-resistance genes are more likely to possess ARGs than bacteria without metal-resistance genes. Metals follow equivalent channels to biocides and antimicrobial compounds, although the proportional quantity of each resource is predicted to fluctuate depending on its predominance in industrial, agricultural, or wastewater. The environmental significance of these dispersed and targeted supplies of metals for testing tolerance in the ecosystem is currently unexplained.

## 5. Antibiotic-Related Environmental Transmission Networks

### 5.1. Wastewater

It is estimated that a significant amount of the medication held by users is expelled in the activated form in fecal matter [114]. Human-ejected antibiotics would reach wastewater treatment systems and appear to have one of three outcomes: biological conversion [115], incorporation into sewerage sludge [116], or unmodified discharge into effluent [117]. For example, antibiotics have been found in groundwater sediments and deposits of the Liuxi River in Guangzhou, China, and fishponds have been suspected to be reservoirs of antibiotic metabolites and resistant genes [118]. Due to the propensity of oxytetracycline to bind to the sludge, it was observed in quite substantial proportions in the sludge compared to various drugs in the influent (1.15–43 mg/kg) [119]. Selective elimination of certain antibiotics, such as sulfonamides [120] and ciprofloxacin [121], from the sludge indicates that the hazards of sludge implementation in the soil appear to be different from the hazards of wastewater effluent disposal in river systems.

### 5.2. Veterinary and Livestock

Animal fecal matter has been found to contaminate the ecosystem with drug-tolerant bacteria and antibiotics [122]. Whenever livestock ingest antibiotics, about 30% to 90% is excreted into urine and feces, exactly as happens among humans [123]. The most prevalent antibiotics recovered were oxytetracycline, doxycycline, and sulfadiazine, preceded by tetracycline, flumequine, lincomycin, and tylosin. A third of feces collections have been reported to contain more than one drug, with swine feces having up to three antibiotics and cow feces having up to eight antibiotics [103]. The spread of AMR on farms has been demonstrated in research in a variety of livestock, particularly swine [124].

### 5.3. Manure and Sludge

The antibiotics discovered in the sludge are mainly less permeable to water, such as norfloxacin, ofloxacin, ciprofloxacin, trimethoprim, sulfamethoxazole, and doxycycline [125]. The amounts of antibiotics in the sludge and compost differ significantly depending on the sources of the influent, the processing setup, the characteristics of antibiotic splitting, and the environmental parameters [126]. In an evaluation of the presence of antibiotics and self-hygiene products in sewage sludge in the United States, two biocides, triclocarban and triclosan (antimicrobial, antifungal), were by far the most prominent analytes, with concentrations as high as 48.1 and 19.7 mg per kg (dry weight). Antibiotics were the next most widespread group of drugs, with levels ranging between 6.8 2.3 and 0.8 0.2 mg per kg (dry weight). The order from highest to lowest rank is ciprofloxacin, ofloxacin, 4-epitetracycline, tetracycline, minocycline, doxycycline, and azithromycin. Interestingly, the biocides and antibiotics evaluated by McClellan and Halden in 2010 [127] fell into a narrow class of antibiotics and biocides with strong selectivity for absorption into wastewater sludge [128].

## 6. Methods of Monitoring AMR

Various methods have been extensively utilized for the monitoring of AMR in the environment. Some of these methods, i.e., culture-based, molecular-based, and nanotechnology-based, are discussed in the following.

### 6.1. Culture-Based Methods

The microbe culture medium (solid/semisolid/broth) in which microbes are grown and quantified in research experiments has traditionally been considered the gold-standard methodology for the detection of antimicrobial-resistant bacteria. Culture-based techniques are inexpensive, relatively precise, and convenient. Antimicrobial-resistant microorganisms can be effectively obtained from samples by incorporating antimicrobials of interest in the growth medium for selection, and if analogous experiments are performed lacking antimicrobials, the fraction of a microbial species that appears to be tolerant can be assessed. For certain antibiotics, such as colistin, the broth microdilution minimum inhibitory concentration (MIC) test is still considered the gold standard for determining sensitivity.

Although this method of AMR surveillance is widely adopted, it has enormous constraints. Many pathogens encountered in the habitat cannot be cultivated under laboratory conditions, false-negative results can arise from samples contaminated with high concentrations of chemicals, and the cultivation phase can be time-consuming, demanding prolonged incubation, numerous steps, and validation assays. The methodology adopted to preserve the specimens, as well as the preservation period, could have a significant impact on the rescue and enumeration of specific entities. Undoubtedly, the major drawback of these techniques is the relatively limited productivity.

Various culture-based automation technologies have been introduced to accelerate lab cultivation and assessment. For example, the VITEK^®^ system [129] (BioMérieux), the MicroScan-Walkaway system [130] (Beckman Coulter), the BD Phoenix^™^ (Becton, Dickinson and Company) [131], and The Biolog Microbial Identification [132] (Biolog inc). These technologies work by testing cultivated organisms in miniature growth chambers filled with various chemicals; any growth or change in colors will be detected and through an algorithm, the strain and its phenotypic characteristics can be obtained. The result is reported as ‘ID’ at the species level accompanied by its antibiotic-sensitivity profile (AST). Usually, these systems require a pure isolate and an incubation period of up to 48 h, and it costs more than the gold-standard biochemical phenotypic testing.

### 6.2. Molecular

To genetically analyze pathogenic and commensal microbial populations, molecular methodologies were implemented. These have been utilized to recognize and monitor antimicrobial-resistant genes. Known ARGs, molecular variables used for the classification of genus and species such as 16s rRNA, along with mobile genetic elements such as integrons (In), insertion sequences (IS), or plasmid-associated genes, which were typically coupled to HGT, are notable examples of targets.

The nucleic acid amplification test (NAAT) is based on the polymerase chain reaction (PCR) that can assess the existence or omission of a targeted gene. In any given sample, ARGs of interest can be detected using specific DNA probes in a conventional PCR experiment. Quantitative PCR (qPCR) provides real-time results and precise statistical outcomes with response rates that are faster than conventional PCR. Using qPCR is a useful technology to investigate the effectiveness of actions on AMR [133]. EpicPCR is the third method with high throughput that allows analysis of entire populations in a strategy that integrates 16s and ARG of each cell, allowing tolerance to be attributed to a particular microbe [134]. The most advanced probe-based PCR method for studying the presence of ARGs of interest in a defined sample is metagenomics, in which a whole sample of DNA collected from an ecological sample can be thoroughly analyzed. This methodology has often been used to recognize genes in a variety of feces samples from people and livestock, including sewage and wastewater effluent [135], medical debris [136], and human and animal guts [137].

Molecular techniques based on PCR are rapid compared to culture-based approaches, and thus can uncover various ARGs, even in bacteria that were challenging to cultivate in laboratories [138]. However, it has been found that the detection of the target sequence typically signifies resistance, but it is essential to illustrate that recognition of the gene is usually not synonymous with tolerance, as demonstrated by diagnostic labs, since genes are not always expressed [139,140]. Currently, many commercial automated PCR-based technologies for bacterial identification (ID) and ARGs are available and widely adopted. These come in cartridges that are preloaded with DNA probes for detection 16s and many ARGs. The tests are rapid (2–6 h) and the samples are loaded directly without the need to isolate and purify the bacteria. These automated systems are expensive, usually not aimed at food and environmental samples, and require continuous updates from the factory to cover new ARGs. Examples of these technologies are GeneXpert^®^ [141] (Cephid) and AMPLICOR^®^ (ROCH), both of which depend on multiplex qPCR technology [142].

The most precise and accurate molecular tool for studying a specific organism and its ARGs to date is the utilization of whole-genome sequencing (WGS). Here, the whole DNA of an organism can be screened, along with ARGs, their copy numbers, mutations, and novel resistance genes. The most used platforms for WGS worldwide are Illumina [143] (Illumina Inc.), Nanopore MinIOn [144] (oxford nanopore technology), and PacBio^®^ HiFi technology [145] (Pacific Bioscience). WGS requires highly skilled technicians, expensive settings, and knowledge in data processing and manipulation. The use of WGS is not considered the first step in active AMR surveillance, but can provide significant information about the source of ARGs and their dynamics, hence they are better suited for use in academic settings or national public health authorities.

### 6.3. Mass Spectrometry

Recently, protein profiling of organisms using mass spectroscopy techniques has been implemented for bacterial identification. Specifically, matrix-assisted laser desorption ionization time-of-flight mass spectrometry (MALDI-ToF MS) is a recent molecular technique that evaluates AMR in microbial specimens. Identifying bacteria with MALDI-ToF is rapid, very accurate, and can process larger volumes than regular molecular techniques. Unfortunately, it requires an isolated bacterium and access to a specialized database, and the initial setup investment is costly. However, many advances have been made in the detection of ARGs using MALDI-ToF instruments, and the machine has been adopted by many hospitals around the world. The two major brands of MALDI-ToF technology in microbial identification are MALDI Biotyper^®^ (Bruker) [146] and VITEK^®^ MS [147] (bioMérieux).

## 7. Current Knowledge Gaps in Understanding AMR

There are still deficits in the literature on the interrelationship between antimicrobial administration in food-producing animals, susceptibility in the biosphere, possible detrimental consequences on people and livestock welfare, and certain associated environmentally related complications as part of the One Health approach. The evaluation of AMR threats in the community from antimicrobial administration in veterinary health care to people and livestock welfare appears to be problematic due to the heterogeneity of the problem and the lack of relevant data on the processes and routes associated with the genomic, biochemical, and community levels. Furthermore, there is a considerable information barrier on the influence that reception habitat exerts on the fate of AMR, multidrug-resistant bacteria, and ARGs. To adequately analyze the implications of AMR in the environment, it could be preferable if the relative input of the environment versus the influence of different factors had been evaluated with reference to the situation of AMR.

In contrast to country funding for research, the Joint Programming Initiative on Antimicrobial Resistance (JPIAMR) is implementing a collaborative strategy in the European Council with the objective of integrating regional research initiatives to better combat the threat of AMR. Network mapping describes countless resources and propagation mechanisms, all of which are presumably driven by the ever-increasing incidence of AMR in therapeutic, animal, and ecological contexts. In addition, the Combating Antibiotic Resistant Bacteria Biopharmaceutical Accelerator (CARB-X) offers financial support to companies that are developing novel and potentially effective solutions to antibiotic resistance [148]. However, more research is required to obtain new insights into the fundamental mechanisms of resistance, gene transfer, and bacterial evolution. This involves an active examination of the function of persistence and host–pathogen interactions, as well as their contribution to antimicrobial resistance. Exploring topics such as these may lead to the discovery of novel therapeutic and diagnostic targets. Furthermore, to adequately understand and address the nascent hazard of AMR, it seems that there is a significant need for political will to carry out novel projects, expand tools, and evaluate risk-analysis strategies.

## 8. Strategies and Action Plan to Combat Antimicrobial Resistance

In April 2014, the World Health Organization recognized AMR as a “significant universal challenge.” The World Health Assembly, the governing body of all WHO member nations, then announced the Global Action Plan on Antimicrobial Resistance (World Health Organization 2015) [149], encouraging member countries to adopt comparable national action plans by May 2017. Various international and national representatives have implemented measures to restrict the prevalence and transmission of antimicrobial resistance by enforcing the appropriate use of antimicrobial medications. The US Food and Drug Administration officially announced measures to evaluate AMR outbreaks [150]. AMR has been substantially suppressed in nations that have implemented synchronized national strategies. Appropriate drug use, antimicrobial surveillance through the One Health approach, improvements in medical practices, the introduction of medical coverage schemes, restricted drug commercialization, a coordinated epidemic management program, and communal management programs all appear to be major determinants in overcoming threats associated with AMR.

A major drawback in the fight against AMR is rapid diagnostic testing, an additional pressing issue, especially in underdeveloped nations where conventional microbiological technologies have been routinely used to identify pathogens. These inadequacies could be resolved by creating custom therapeutics for appropriate antimicrobial treatment based on modern and advanced genomic screening technologies. A One-Health strategy would perhaps represent a significant approach for exploring the human–animal interplay and introducing innovative assessment tactics. Taking into account the routes of transmission of antimicrobial resistance that have been known to be prevalent among the three elements, including people, animals, and the environment [151], these areas of research are of special relevance and must be addressed effectively. However, the indiscriminate and illogical use of drugs is a major contributor to AMR, specifically in low- and middle-income countries. Antimicrobial agents are used inappropriately for a variety of purposes, including inpatient treatment with prescribed medications by physicians, lack of knowledge of antibiotics, inaccurate prognosis, especially in emerging countries, and distressing hardships for clinicians imposed primarily by the pharmaceutical industry.

A limitation of innovative drugs mainly hampers the analysis of the whole issue of AMR [152]; therefore, breakthroughs in antibiotic discovery, combination therapy, and technological innovation [153] are required. Future analysis will focus on identifying the adverse effects of human activity, the participation of various major determinants of AMR, the consequences of resistant strains on human and animal wellness, and significant technical, cultural, and financial approaches to reduce environmental resistance to antimicrobials. Various plans, such as the United States National Action Plan to Combat Antimicrobial Resistant Bacteria (White House 2015), the declaration of the 2016 high-level meeting on antimicrobial resistance at the United Nations General Assembly (OPGA/WHO/FAO/OIE 2016), and the FAO/OIE/WHO Tripartite Collaboration are current national and international approaches to resolve the spread of AMR.

## 9. Conclusions

ARGs possess the ability to transition from bacterial populations between human and livestock infections, and conversely, since gene exchange among bacteria might happen anywhere theoretically, it is particularly prone to occur among taxonomically highly associated bacteria. Furthermore, in reality, to transfer resistance, host and recipient bacteria must occupy an equivalent biological habitat, at least for a short period. Taking this into account, it makes perfect sense that the prevalence of resistance transmission that occurs between animal- and human-associated microbes should be stronger. The true consequence of either of such environmental circumstances appears to be unclear because of the limited knowledge of the mechanisms that cause the transference of ARG in bacterial populations and the subsequent persistence functionality of ARG or host bacteria when transported under such circumstances.

In addition to the increasing international recognition of antimicrobial resistance, we still have significant gaps in our awareness in terms of the prevalence, location, and drivers of AMR at the community level. To increase our knowledge of the severity of AMR in communities, as well as the population-level variables that impact the establishment and distribution of AMR, we must incorporate a wide range of context-specific epidemiological research methodologies. A comprehensive and cost-effective AMR approach must incorporate a multidisciplinary framework to reduce antibiotic consumption, improve monitoring and control, and strengthen the user and general practitioner in the administration of antibiotics. Although the existing severity of the situation and its distribution in both the population and hospitals complicate the situation, it is essential, as health-care personnel contribute greatly to minimizing the genesis and transmission of resistance. This research, in collaboration with management programs, will help find approaches to the recognition, mitigation, and monitoring of AMR, as well as the prolonged use of antimicrobials in the treatment regime.

## Data Availability

The data are contained within the article.

## References

[1] Antimicrobial Resistance. https://www.who.int/news-room/fact-sheets/detail/antimicrobial-resistance.

[2] D’Costa V.M., King C.E., Kalan L., Morar M., Sung W.W.L., Schwarz C., Froese D., Zazula G., Calmels F., Debruyne R. (2011). Antibiotic Resistance Is Ancient. Nature.

[3] Holmes A.H., Moore L.S.P., Sundsfjord A., Steinbakk M., Regmi S., Karkey A., Guerin P.J., Piddock L.J.V. (2016). Understanding the Mechanisms and Drivers of Antimicrobial Resistance. Lancet.

[4] Richardson J., Lockhart C., Pongolini S., Karesh W.B., Baylis M., Goldberg T., Slingenbergh J., Gale P., Venturini T., Catchpole M. (2016). Drivers for Emerging Issues in Animal and Plant Health. EFSA J..

[5] Zinsstag J. (2012). Convergence of Ecohealth and One Health. EcoHealth.

[6] Collignon P.J., McEwen S.A. (2019). One Health—Its Importance in Helping to Better Control Antimicrobial Resistance. Trop. Med. Infect. Dis..

[7] Trotter A.J., Aydin A., Strinden M.J., O’Grady J. (2019). Recent and Emerging Technologies for the Rapid Diagnosis of Infection and Antimicrobial Resistance. Curr. Opin. Microbiol..

[8] Antimicrobial Resistance: Tackling a Crisis for the Health and Wealth of Nations/the Review on Antimicrobial Resistance Chaired by Jim O’Neill. https://wellcomecollection.org/works/rdpck35v.

[9] Murray C.J., Ikuta K.S., Sharara F., Swetschinski L., Aguilar G.R., Gray A., Han C., Bisignano C., Rao P., Wool E. (2022). Global Burden of Bacterial Antimicrobial Resistance in 2019: A Systematic Analysis. Lancet.

[10] New Report Calls for Urgent Action to Avert Antimicrobial Resistance Crisis. https://www.who.int/news/item/29-04-2019-new-report-calls-for-urgent-action-to-avert-antimicrobial-resistance-crisis.

[11] Davies J., Davies D. (2010). Origins and Evolution of Antibiotic Resistance. Microbiol. Mol. Biol. Rev. MMBR.

[12] Meletis G. (2016). Carbapenem Resistance: Overview of the Problem and Future Perspectives. Ther. Adv. Infect. Dis..

[13] Du H., Chen L., Tang Y.-W., Kreiswirth B.N. (2016). Emergence of the Mcr-1 Colistin Resistance Gene in Carbapenem-Resistant Enterobacteriaceae. Lancet Infect. Dis..

[14] Yao X., Doi Y., Zeng L., Lv L., Liu J.-H. (2016). Carbapenem-Resistant and Colistin-Resistant *Escherichia Coli* Co-Producing NDM-9 and MCR-1. Lancet Infect. Dis..

[15] Savin M., Bierbaum G., Blau K., Parcina M., Sib E., Smalla K., Schmithausen R., Heinemann C., Hammerl J.A., Kreyenschmidt J. (2020). Colistin-Resistant Enterobacteriaceae Isolated From Process Waters and Wastewater From German Poultry and Pig Slaughterhouses. Front. Microbiol..

[16] Hassuna N.A., AbdelAziz R.A., Zakaria A., Abdelhakeem M. (2020). Extensively-Drug Resistant *Klebsiella Pneumoniae* Recovered From Neonatal Sepsis Cases From a Major NICU in Egypt. Front. Microbiol..

[17] Ashiru-Oredope D., Kessel A., Hopkins S., Ashiru-Oredope D., Brown B., Brown N., Carter S., Cichowka A., Susan Hopkins on behalf of the English Surveillance Programme for Antimicrobial Utilization and Resistance Oversight Group (2013). Antimicrobial Stewardship: English Surveillance Programme for Antimicrobial Utilization and Resistance (ESPAUR). J. Antimicrob. Chemother..

[18] Sipahi O.R. (2008). Economics of Antibiotic Resistance. Expert Rev. Anti Infect. Ther..

[19] Taylor J., Hafner M., Yerushalmi E., Smith R., Bellasio J., Vardavas R., Bienkowska-Gibbs T., Rubin J. Estimating the Economic Costs of Antimicrobial Resistance: Model and Results. https://www.rand.org/pubs/research_reports/RR911.html.

[20] Devasahayam G., Scheld W.M., Hoffman P.S. (2010). Newer Antibacterial Drugs for a New Century. Expert Opin. Investig. Drugs.

[21] Garcia-Migura L., Hendriksen R.S., Fraile L., Aarestrup F.M. (2014). Antimicrobial Resistance of Zoonotic and Commensal Bacteria in Europe: The Missing Link between Consumption and Resistance in Veterinary Medicine. Vet. Microbiol..

[22] Marshall B.M., Levy S.B. (2011). Food Animals and Antimicrobials: Impacts on Human Health. Clin. Microbiol. Rev..

[23] Woolhouse M., Ward M., van Bunnik B., Farrar J. (2015). Antimicrobial Resistance in Humans, Livestock and the Wider Environment. Philos. Trans. R. Soc. B Biol. Sci..

[24] Finley R.L., Collignon P., Larsson D.G.J., McEwen S.A., Li X.-Z., Gaze W.H., Reid-Smith R., Timinouni M., Graham D.W., Topp E. (2013). The Scourge of Antibiotic Resistance: The Important Role of the Environment. Clin. Infect. Dis..

[25] Tackling Drug-Resistant Infections Globally: An Overview of Our Work/the Review on Antimicrobial Resistance Chaired by Jim O’Neill. https://wellcomecollection.org/works/e8njjeed.

[26] Weir M., Rajić A., Dutil L., Uhland C., Bruneau N. Zoonotic Bacteria and Antimicrobial Resistance in Aquaculture: Opportunities for Surveillance in Canada. https://www.ncbi.nlm.nih.gov/pmc/articles/PMC3354819/.

[27] Klous G., Huss A., Heederik D.J.J., Coutinho R.A. (2016). Human–Livestock Contacts and Their Relationship to Transmission of Zoonotic Pathogens, a Systematic Review of Literature. One Health.

[28] Starr M.P., Reynolds D.M. (1951). Streptomycin Resistance of Coliform Bacteria from Turkeys Fed Streptomycin. Am. J. Public Health Nations Health.

[29] Liu Y.-Y., Wang Y., Walsh T.R., Yi L.-X., Zhang R., Spencer J., Doi Y., Tian G., Dong B., Huang X. (2016). Emergence of Plasmid-Mediated Colistin Resistance Mechanism *mcr-1* in Animals and Human Beings in China: A Microbiological and Molecular Biological Study. Lancet Infect. Dis..

[30] Rhouma M., Beaudry F., Thériault W., Letellier A. (2016). Colistin in Pig Production: Chemistry, Mechanism of Antibacterial Action, Microbial Resistance Emergence, and One Health Perspectives. Front. Microbiol..

[31] Baron S., Hadjadj L., Rolain J.-M., Olaitan A.O. (2016). Molecular Mechanisms of Polymyxin Resistance: Knowns and Unknowns. Int. J. Antimicrob. Agents.

[32] Karakonstantis S., Kritsotakis E.I., Gikas A. (2020). Pandrug-Resistant Gram-Negative Bacteria: A Systematic Review of Current Epidemiology, Prognosis and Treatment Options. J. Antimicrob. Chemother..

[33] Voss A., Loeffen F., Bakker J., Klaassen C., Wulf M. (2005). Methicillin-Resistant *Staphylococcus Aureus* in Pig Farming. Emerg. Infect. Dis..

[34] Wendlandt S., Kadlec K., Feßler A.T., Monecke S., Ehricht R., van de Giessen A.W., Hengeveld P.D., Huijsdens X., Schwarz S., van Duijkeren E. (2013). Resistance Phenotypes and Genotypes of Methicillin-Resistant *Staphylococcus Aureus* Isolates from Broiler Chickens at Slaughter and Abattoir Workers. J. Antimicrob. Chemother..

[35] Kadlec K., Ehricht R., Monecke S., Steinacker U., Kaspar H., Mankertz J., Schwarz S. (2009). Diversity of Antimicrobial Resistance Pheno- and Genotypes of Methicillin-Resistant *Staphylococcus Aureus* ST398 from Diseased Swine. J. Antimicrob. Chemother..

[36] Köck R., Schaumburg F., Mellmann A., Köksal M., Jurke A., Becker K., Friedrich A.W. (2013). Livestock-Associated Methicillin-Resistant *Staphylococcus Aureus* (MRSA) as Causes of Human Infection and Colonization in Germany. PLoS ONE.

[37] Prestinaci F., Pezzotti P., Pantosti A. (2015). Antimicrobial Resistance: A Global Multifaceted Phenomenon. Pathog. Glob. Health.

[38] Melese A., Genet C., Andualem T. (2020). Prevalence of Vancomycin Resistant *Enterococci* (VRE) in Ethiopia: A Systematic Review and Meta-Analysis. BMC Infect. Dis..

[39] Werner G., Coque T.M., Hammerum A.M., Hope R., Hryniewicz W., Johnson A., Klare I., Kristinsson K.G., Leclercq R., Lester C.H. (2008). Emergence and Spread of Vancomycin Resistance among *Enterococci* in Europe. Euro Surveill. Bull. Eur. Sur Mal. Transm. Eur. Commun. Dis. Bull..

[40] Bourgeois-Nicolaos N., Moubareck C., Mangeney N., Butel M.-J., Doucet-Populaire F. (2006). Comparative Study of *VanA* Gene Transfer from *Enterococcus Faecium* to *Enterococcus Faecalis* and to *Enterococcus Faecium* in the Intestine of Mice. FEMS Microbiol. Lett..

[41] Arias C.A., Murray B.E. (2012). The Rise of the *Enterococcus*: Beyond Vancomycin Resistance. Nat. Rev. Microbiol..

[42] Dadashi M., Sharifian P., Bostanshirin N., Hajikhani B., Bostanghadiri N., Khosravi-Dehaghi N., van Belkum A., Darban-Sarokhalil D. (2021). The Global Prevalence of Daptomycin, Tigecycline, and Linezolid-Resistant *Enterococcus Faecalis* and *Enterococcus Faecium* Strains from Human Clinical Samples: A Systematic Review and Meta-Analysis. Front. Med..

[43] Wang Z., He J., Li Q., Tang Y., Wang J., Pan Z., Chen X., Jiao X. (2020). First Detection of NDM-5-Positive *Salmonella Enterica* Serovar Typhimurium Isolated from Retail Pork in China. Microb. Drug Resist. Larchmt. N.

[44] Díaz M.Á., Díaz P.L., Rodríguez E.C., Montaño L.A., Gartner D.M., Vernaza M.E., Eljach V., Realpe M.E. (2013). A nalidixic acid-resistant Salmonella enteritidis outbreak in Popayán, Cauca, 2011. Biomed. Rev. Inst. Nac. Salud.

[45] Zhao L., Dong Y.H., Wang H. (2010). Residues of Veterinary Antibiotics in Manures from Feedlot Livestock in Eight Provinces of China. Sci. Total Environ..

[46] Otto S.J.G., Carson C.A., Finley R.L., Thomas M.K., Reid-Smith R.J., McEwen S.A. (2014). Estimating the Number of Human Cases of Ceftiofur-Resistant *Salmonella Enterica* Serovar Heidelberg in Québec and Ontario, Canada. Clin. Infect. Dis..

[47] Smith K.E., Besser J.M., Hedberg C.W., Leano F.T., Bender J.B., Wicklund J.H., Johnson B.P., Moore K.A., Osterholm M.T. (1999). Quinolone-Resistant *Campylobacter Jejuni* Infections in Minnesota, 1992-1998. Investigation Team. N. Engl. J. Med..

[48] Capozzi C., Volpi A., Maurici M., Lisena F.P., Visconti G., Panà A. (2013). Healthcare-associated infections and antibiotic resistance: A global challenge for the 21st century. Ig. Sanita Pubblica.

[49] Levy S.B. (1998). The Challenge of Antibiotic Resistance. Sci. Am..

[50] Crofts T.S., Gasparrini A.J., Dantas G. (2017). Next-Generation Approaches to Understand and Combat the Antibiotic Resistome. Nat. Rev. Microbiol..

[51] Forsberg K.J., Patel S., Wencewicz T.A., Dantas G. (2015). The Tetracycline Destructases: A Novel Family of Tetracycline-Inactivating Enzymes. Chem. Biol..

[52] Shaw W.V., Packman L.C., Burleigh B.D., Dell A., Morris H.R., Hartley B.S. (1979). Primary Structure of a Chloramphenicol Acetyltransferase Specified by R Plasmids. Nature.

[53] Yang W., Moore I.F., Koteva K.P., Bareich D.C., Hughes D.W., Wright G.D. (2004). TetX Is a Flavin-Dependent Monooxygenase Conferring Resistance to Tetracycline Antibiotics. J. Biol. Chem..

[54] Bush K. (2010). Bench-to-Bedside Review: The Role of Beta-Lactamases in Antibiotic-Resistant Gram-Negative Infections. Crit. Care Lond. Engl..

[55] Piddock L.J.V. (2006). Clinically Relevant Chromosomally Encoded Multidrug Resistance Efflux Pumps in Bacteria. Clin. Microbiol. Rev..

[56] Roberts M.C. (1996). Tetracycline Resistance Determinants: Mechanisms of Action, Regulation of Expression, Genetic Mobility, and Distribution. FEMS Microbiol. Rev..

[57] On the Mechanism of Solute Uptake in Pseudomonas—PubMed. https://pubmed.ncbi.nlm.nih.gov/12700103/.

[58] Babouee Flury B., Ellington M.J., Hopkins K.L., Turton J.F., Doumith M., Loy R., Staves P., Hinic V., Frei R., Woodford N. (2016). Association of Novel Nonsynonymous Single Nucleotide Polymorphisms in AmpD with Cephalosporin Resistance and Phylogenetic Variations in AmpC, AmpR, OmpF, and OmpC in *Enterobacter Cloacae* Isolates That Are Highly Resistant to Carbapenems. Antimicrob. Agents Chemother..

[59] Understanding Antibiotic Resistance. https://www.open.edu/openlearn/mod/oucontent/science-maths-technology/understanding-antibiotic-resistance.

[60] D’Costa V.M., McGrann K.M., Hughes D.W., Wright G.D. (2006). Sampling the Antibiotic Resistome. Science.

[61] Vikesland P.J., Pruden A., Alvarez P.J.J., Aga D., Bürgmann H., Li X.-D., Manaia C.M., Nambi I., Wigginton K., Zhang T. (2017). Toward a Comprehensive Strategy to Mitigate Dissemination of Environmental Sources of Antibiotic Resistance. Environ. Sci. Technol..

[62] Vikesland P., Garner E., Gupta S., Kang S., Maile-Moskowitz A., Zhu N. (2019). Differential Drivers of Antimicrobial Resistance across the World. Acc. Chem. Res..

[63] Payne D.J., Miller L.F., Findlay D., Anderson J., Marks L. (2015). Time for a Change: Addressing R&D and Commercialization Challenges for Antibacterials. Philos. Trans. R. Soc. B Biol. Sci..

[64] Klein E.Y., Van Boeckel T.P., Martinez E.M., Pant S., Gandra S., Levin S.A., Goossens H., Laxminarayan R. (2018). Global Increase and Geographic Convergence in Antibiotic Consumption between 2000 and 2015. Proc. Natl. Acad. Sci. USA.

[65] ResistanceMap—Antibiotic Use. https://resistancemap.cddep.org/.

[66] Gasson J., Blockman M., Willems B. (2018). Antibiotic Prescribing Practice and Adherence to Guidelines in Primary Care in the Cape Town Metro District, South Africa. S. Afr. Med. J..

[67] Sarwar M.R., Saqib A., Iftikhar S., Sadiq T. (2018). Antimicrobial Use by WHO Methodology at Primary Health Care Centers: A Cross Sectional Study in Punjab, Pakistan. BMC Infect. Dis..

[68] Wang J., Wang P., Xinghe W., Zheng Y., Xiao Y. (2014). Use and Prescription of Antibiotics in Primary Health Care Settings in China. JAMA Intern. Med..

[69] Schwartz K.L., Langford B.J., Daneman N., Chen B., Brown K.A., McIsaac W., Tu K., Candido E., Johnstone J., Leung V. (2020). Unnecessary Antibiotic Prescribing in a Canadian Primary Care Setting: A Descriptive Analysis Using Routinely Collected Electronic Medical Record Data. CMAJ Open.

[70] Richard V.M., Jonathan K.W., Jonathan E., Jonathan H., Pedro C., Jefferson B. (2019). Reducing Inappropriate Outpatient Antibiotic Prescribing: Normative Comparison Using Unblinded Provider Reports. BMJ Open Qual..

[71] Kariyawasam R.M., Julien D.A., Jelinski D.C., Larose S.L., Rennert-May E., Conly J.M., Dingle T.C., Chen J.Z., Tyrrell G.J., Ronksley P.E. (2022). Antimicrobial Resistance (AMR) in COVID-19 Patients: A Systematic Review and Meta-Analysis (November 2019–June 2021). Antimicrob. Resist. Infect. Control.

[72] WHO Publishes List of Bacteria for Which New Antibiotics Are Urgently Needed. https://www.who.int/news/item/27-02-2017-who-publishes-list-of-bacteria-for-which-new-antibiotics-are-urgently-needed.

[73] National Research Council (US), Committee to Study the Human Health Effects of Subtherapeutic Antibiotic Use in Animal Feeds (1980). The Effects on Human Health of Subtherapeutic Use of Antimicrobials in Animal Feeds.

[74] Fifth OIE Annual Report on Antimicrobial Agents Intended for Use in Animals. https://www.woah.org/en/document/fifth-oie-annual-report-on-antimicrobial-agents-intended-for-use-in-animals/.

[75] Tiseo K., Huber L., Gilbert M., Robinson T.P., Van Boeckel T.P. (2020). Global Trends in Antimicrobial Use in Food Animals from 2017 to 2030. Antibiotics.

[76] Regulation (EC) No 1831/2003 of the European Parliament and of the Council. https://eur-lex.europa.eu/eli/reg/2003/1831/oj/eng.

[77] U.S. Food and Drug Administration. Food Code 2013. https://www.fda.gov/food/fda-food-code/food-code-2013.

[78] Ejo M., Garedew L., Alebachew Z., Worku W. (2016). Prevalence and Antimicrobial Resistance of *Salmonella* Isolated from Animal-Origin Food Items in Gondar, Ethiopia. BioMed Res. Int..

[79] Rasheed M.U., Thajuddin N., Ahamed P., Teklemariam Z., Jamil K. (2014). Antimicrobial Drug Resistance in Strains of *Escherichia Coli* Isolated from Food Sources. Rev. Inst. Med. Trop. São Paulo.

[80] Kahrilas G.A., Blotevogel J., Stewart P.S., Borch T. (2015). Biocides in Hydraulic Fracturing Fluids: A Critical Review of Their Usage, Mobility, Degradation, and Toxicity. Environ. Sci. Technol..

[81] Buffet-Bataillon S., Tattevin P., Bonnaure-Mallet M., Jolivet-Gougeon A. (2012). Emergence of Resistance to Antibacterial Agents: The Role of Quaternary Ammonium Compounds—A Critical Review. Int. J. Antimicrob. Agents.

[82] Poole K. (2002). Mechanisms of Bacterial Biocide and Antibiotic Resistance. J. Appl. Microbiol..

[83] Hugo W.B., Longworth A.R. (1964). Effect of Chlorhexidine Diacetate on “Protoplasts” and Spheroplasts of *Escherichia Coli*, Protoplasts of *Bacillus Megaterium* and the Gram Staining Reaction of *Staphylococcus Aureus*. J. Pharm. Pharmacol..

[84] McDonnell G., Russell A.D. (1999). Antiseptics and Disinfectants: Activity, Action, and Resistance. Clin. Microbiol. Rev..

[85] Russell A.D. (1995). Mechanisms of Bacterial Resistance to Biocides. Int. Biodeterior. Biodegrad..

[86] Brown M.R., Gilbert P. (1993). Sensitivity of Biofilms to Antimicrobial Agents. J. Appl. Bacteriol..

[87] McMurry L.M., McDermott P.F., Levy S.B. (1999). Genetic Evidence That InhA of *Mycobacterium Smegmatis* Is a Target for Triclosan. Antimicrob. Agents Chemother..

[88] Sasatsu M., Shirai Y., Hase M., Noguchi N., Kono M., Behr H., Freney J., Arai T. (1995). The Origin of the Antiseptic-Resistance Gene Ebr in *Staphylococcus Aureus*. Microbios.

[89] Russell A.D. (1997). Plasmids and Bacterial Resistance to Biocides. J. Appl. Microbiol..

[90] Prince H.N., Nonemaker W.S., Norgard R.C., Prince D.L. (1978). Drug Resistance Studies with Topical Antiseptics. J. Pharm. Sci..

[91] Fitzgerald K.A., Davies A., Russell A.d. (1992). Sensitivity and Resistance of *Escherichia Coli* and *Staphylococcus Aureus* to Chlorhexidine. Lett. Appl. Microbiol..

[92] Silver S., Misra T.K. (1988). Plasmid-Mediated Heavy Metal Resistances. Annu. Rev. Microbiol..

[93] Vijayakumar R., Sandle T. (2019). A Review on Biocide Reduced Susceptibility Due to Plasmid-Borne Antiseptic-Resistant Genes—Special Notes on Pharmaceutical Environmental Isolates. J. Appl. Microbiol..

[94] Regulation (EU) No 528/2012 of the European Parliament and of the Council of 22 May 2012 Concerning the Making. Market and Use of Biocidal Products Text with EEA Relevance. 2012. L 167/1, pp. 1–123. https://www.legislation.gov.uk/eur/2012/528/contents.

[95] Sattar S.A., Tetro J.A., Springthorpe V.S. (2007). Effects of Environmental Chemicals and the Host-Pathogen Relationship: Are There Any Negative Consequences for Human Health?. New Biocides Development.

[96] Karvelas M., Katsoyiannis A., Samara C. (2003). Occurrence and Fate of Heavy Metals in the Wastewater Treatment Process. Chemosphere.

[97] Nicholson F.A., Smith S.R., Alloway B.J., Carlton-Smith C., Chambers B.J. (2003). An Inventory of Heavy Metals Inputs to Agricultural Soils in England and Wales. Sci. Total Environ..

[98] Baker-Austin C., Wright M.S., Stepanauskas R., McArthur J.V. (2006). Co-Selection of Antibiotic and Metal Resistance. Trends Microbiol..

[99] Nies D.H. (2003). Efflux-Mediated Heavy Metal Resistance in Prokaryotes. FEMS Microbiol. Rev..

[100] Ardestani M.M., van Straalen N.M., van Gestel C.A.M. (2015). Biotic Ligand Modeling Approach: Synthesis of the Effect of Major Cations on the Toxicity of Metals to Soil and Aquatic Organisms. Environ. Toxicol. Chem..

[101] Olaniran A.O., Balgobind A., Pillay B. (2013). Bioavailability of Heavy Metals in Soil: Impact on Microbial Biodegradation of Organic Compounds and Possible Improvement Strategies. Int. J. Mol. Sci..

[102] Tipping E., Lofts S. (2013). Metal Mixture Toxicity to Aquatic Biota in Laboratory Experiments: Application of the WHAM-FTOX Model. Aquat. Toxicol..

[103] Singer A.C., Shaw H., Rhodes V., Hart A. (2016). Review of Antimicrobial Resistance in the Environment and Its Relevance to Environmental Regulators. Front. Microbiol..

[104] Giller K.E., Witter E., Mcgrath S.P. (1998). Toxicity of Heavy Metals to Microorganisms and Microbial Processes in Agricultural Soils: A Review. Soil Biol. Biochem..

[105] Smith S.R. (2009). A Critical Review of the Bioavailability and Impacts of Heavy Metals in Municipal Solid Waste Composts Compared to Sewage Sludge. Environ. Int..

[106] Braam F., Klapwijk A. (1981). Effect of Copper on Nitrification in Activated Sludge. Water Res..

[107] Waara K.-O. (1992). Effects of Copper, Cadmium, Lead and Zinc on Nitrate Reduction in a Synthetic Water Medium and Lake Water from Northern Sweden. Water Res..

[108] Ajmal M., Ahmad A., Nomani A.A. (1983). Influence of Toxic Metals on the Repression of Carbonaceous Oxygen Demand. Water Res..

[109] Chipasa K.B. (2003). Accumulation and Fate of Selected Heavy Metals in a Biological Wastewater Treatment System. Waste Manag..

[110] Zhang C., Nie S., Liang J., Zeng G., Wu H., Hua S., Liu J., Yuan Y., Xiao H., Deng L. (2016). Effects of Heavy Metals and Soil Physicochemical Properties on Wetland Soil Microbial Biomass and Bacterial Community Structure. Sci. Total Environ..

[111] Xie Y., Fan J., Zhu W., Amombo E., Lou Y., Chen L., Fu J. (2016). Effect of Heavy Metals Pollution on Soil Microbial Diversity and Bermudagrass Genetic Variation. Front. Plant Sci..

[112] Jacob J.M., Karthik C., Saratale R.G., Kumar S.S., Prabakar D., Kadirvelu K., Pugazhendhi A. (2018). Biological Approaches to Tackle Heavy Metal Pollution: A Survey of Literature. J. Environ. Manage..

[113] Imran M., Das K.R., Naik M.M. (2019). Co-Selection of Multi-Antibiotic Resistance in Bacterial Pathogens in Metal and Microplastic Contaminated Environments: An Emerging Health Threat. Chemosphere.

[114] Verlicchi P., Zambello E. (2016). Predicted and Measured Concentrations of Pharmaceuticals in Hospital Effluents. Examination of the Strengths and Weaknesses of the Two Approaches through the Analysis of a Case Study. Sci. Total Environ..

[115] Chen C.-E., Zhang H., Ying G.-G., Zhou L.-J., Jones K.C. (2015). Passive Sampling: A Cost-Effective Method for Understanding Antibiotic Fate, Behaviour and Impact. Environ. Int..

[116] Ahmed M.B., Zhou J.L., Ngo H.H., Guo W. (2015). Adsorptive Removal of Antibiotics from Water and Wastewater: Progress and Challenges. Sci. Total Environ..

[117] Luo Y., Guo W., Ngo H.H., Nghiem L.D., Hai F.I., Zhang J., Liang S., Wang X.C. (2014). A Review on the Occurrence of Micropollutants in the Aquatic Environment and Their Fate and Removal during Wastewater Treatment. Sci. Total Environ..

[118] Xiong W., Sun Y., Ding X., Wang M., Zeng Z. (2015). Selective Pressure of Antibiotics on ARGs and Bacterial Communities in Manure-Polluted Freshwater-Sediment Microcosms. Front. Microbiol..

[119] Gardner M., Jones V., Comber S., Scrimshaw M.D., Coello-Garcia T., Cartmell E., Lester J., Ellor B. (2013). Performance of UK Wastewater Treatment Works with Respect to Trace Contaminants. Sci. Total Environ..

[120] Yang S.-F., Lin C.-F., Wu C.-J., Ng K.-K., Yu-Chen Lin A., Andy Hong P.-K. (2012). Fate of Sulfonamide Antibiotics in Contact with Activated Sludge—Sorption and Biodegradation. Water Res..

[121] Polesel F., Lehnberg K., Dott W., Trapp S., Thomas K.V., Plósz B.G. (2015). Factors Influencing Sorption of Ciprofloxacin onto Activated Sludge: Experimental Assessment and Modelling Implications. Chemosphere.

[122] Wichmann F., Udikovic-Kolic N., Andrew S., Handelsman J. (2014). Diverse Antibiotic Resistance Genes in Dairy Cow Manure. mBio.

[123] Berendsen B.J.A., Wegh R.S., Memelink J., Zuidema T., Stolker L.A.M. (2015). The Analysis of Animal Faeces as a Tool to Monitor Antibiotic Usage. Talanta.

[124] Bloom of Resident Antibiotic-Resistant Bacteria in Soil Following Manure Fertilization|PNAS. https://www.pnas.org/content/111/42/15202.

[125] Clarke B.O., Smith S.R. (2011). Review of ‘Emerging’ Organic Contaminants in Biosolids and Assessment of International Research Priorities for the Agricultural Use of Biosolids. Environ. Int..

[126] Li W., Shi Y., Gao L., Liu J., Cai Y. (2013). Occurrence, Distribution and Potential Affecting Factors of Antibiotics in Sewage Sludge of Wastewater Treatment Plants in China. Sci. Total Environ..

[127] McClellan K., Halden R.U. (2010). Pharmaceuticals and Personal Care Products in Archived U.S. Biosolids from the 2001 EPA National Sewage Sludge Survey. Water Res..

[128] Li B., Zhang T. (2010). Biodegradation and Adsorption of Antibiotics in the Activated Sludge Process. Environ. Sci. Technol..

[129] VITEK® 2: Healthcare. https://www.biomerieux-usa.com/vitek-2.

[130] MicroScan WalkAway plus Microbiology System. httpss://www.beckmancoulter.com/products/microbiology/microscan-walkaway-plus-system.

[131] BD PhoenixTM Automated Identification and Susceptibility Testing System. https://www.bd.com/en-ca/offerings/capabilities/microbiology-solutions/identification-and-susceptibility-testing/bd-phoenix-automated-identification-and-susceptibility-testing-system.

[132] Biolog—Microbial Identification & Characterization—Biolog—World Leader in Cell Based Technology and Assays for Microbiology & Cell Biology Using Phenotype Microarray Technology. https://www.biolog.com/.

[133] Tien Y.-C., Li B., Zhang T., Scott A., Murray R., Sabourin L., Marti R., Topp E. (2017). Impact of Dairy Manure Pre-Application Treatment on Manure Composition, Soil Dynamics of Antibiotic Resistance Genes, and Abundance of Antibiotic-Resistance Genes on Vegetables at Harvest. Sci. Total Environ..

[134] Spencer S.J., Tamminen M.V., Preheim S.P., Guo M.T., Briggs A.W., Brito I.L., Weitz D.A., Pitkänen L.K., Vigneault F., Virta M.P. (2016). Massively Parallel Sequencing of Single Cells by EpicPCR Links Functional Genes with Phylogenetic Markers. ISME J..

[135] Guo J., Li J., Chen H., Bond P.L., Yuan Z. (2017). Metagenomic Analysis Reveals Wastewater Treatment Plants as Hotspots of Antibiotic Resistance Genes and Mobile Genetic Elements. Water Res..

[136] Munk P., Andersen V.D., de Knegt L., Jensen M.S., Knudsen B.E., Lukjancenko O., Mordhorst H., Clasen J., Agersø Y., Folkesson A. (2017). A Sampling and Metagenomic Sequencing-Based Methodology for Monitoring Antimicrobial Resistance in Swine Herds. J. Antimicrob. Chemother..

[137] Thomas M., Webb M., Ghimire S., Blair A., Olson K., Fenske G.J., Fonder A.T., Christopher-Hennings J., Brake D., Scaria J. (2017). Metagenomic Characterization of the Effect of Feed Additives on the Gut Microbiome and Antibiotic Resistome of Feedlot Cattle. Sci. Rep..

[138] Luby E., Ibekwe A.M., Zilles J., Pruden A. (2016). Molecular Methods for Assessment of Antibiotic Resistance in Agricultural Ecosystems: Prospects and Challenges. J. Environ. Qual..

[139] M100Ed32|Performance Standards for Antimicrobial Susceptibility Testing, 32nd Edition. https://clsi.org/standards/products/microbiology/documents/m100/.

[140] ISO—ISO 20776-1:2006—Clinical Laboratory Testing and in Vitro Diagnostic Test Systems—Susceptibility Testing of Infectious Agents and Evaluation of Performance of Antimicrobial Susceptibility Test Devices—Part 1: Reference Method for Testing the in Vitro Activity of Antimicrobial Agents against Rapidly Growing Aerobic Bacteria Involved in Infectious Diseases. https://www.iso.org/standard/41630.html.

[141] Cepheid|Enhanced Antibiotic Stewardship. https://www.cepheid.com/en/impact/enhanced-antibiotic-stewardship.

[142] Molecular Diagnostics. https://diagnostics.roche.com/global/en/products/product-category/molecular-diagnostics.html.

[143] Sequencing|Key Methods and Uses. https://www.illumina.com/techniques/sequencing.html.

[144] MinION. http://nanoporetech.com/products/minion.

[145] How HiFi Sequencing Works. https://www.pacb.com/technology/hifi-sequencing/how-it-works/.

[146] Microbial Identification. https://www.bruker.com/en/products-and-solutions/microbiology-and-diagnostics/microbial-identification.html.

[147] VITEK®, MS. https://www.biomerieux-diagnostics.com/Massspectrometrymicrobialidentificationsystem.

[148] Home—Carb-X. https://carb-x.org/.

[149] Global Action Plan on Antimicrobial Resistance. https://www.who.int/publications-detail-redirect/9789241509763.

[150] Huang Q.M., Horn M.A., Ruan S.G. (2019). Modeling the Effect of Antibiotic Exposure on the Transmission of Methicillin-Resistant *Staphylococcus Aureus* in Hospitals with Environmental Contamination. Math. Biosci. Eng. MBE.

[151] Cantón R., Akóva M., Carmeli Y., Giske C.G., Glupczynski Y., Gniadkowski M., Livermore D.M., Miriagou V., Naas T., Rossolini G.M. (2012). Rapid Evolution and Spread of Carbapenemases among Enterobacteriaceae in Europe. Clin. Microbiol. Infect. Off. Publ. Eur. Soc. Clin. Microbiol. Infect. Dis..

[152] Bigdeli M., Jacobs B., Tomson G., Laing R., Ghaffar A., Dujardin B., Van Damme W. (2013). Access to Medicines from a Health System Perspective. Health Policy Plan..

[153] Fischbach M.A. (2011). Combination Therapies for Combating Antimicrobial Resistance. Curr. Opin. Microbiol..

